# Human hypoxic pulmonary vasoconstriction is unaltered by 8 h of preceding isocapnic hyperoxia

**DOI:** 10.14814/phy2.13396

**Published:** 2017-09-12

**Authors:** Hung‐Yuan Cheng, Quentin P. P. Croft, Matthew C. Frise, Nick P. Talbot, Nayia Petousi, Peter A. Robbins, Keith L. Dorrington

**Affiliations:** ^1^ Department of Physiology, Anatomy & Genetics University of Oxford Oxford United Kingdom

**Keywords:** Cardiac output, human, hyperoxia, pulmonary circulation

## Abstract

Exposure to sustained hypoxia of 8 h duration increases the sensitivity of the pulmonary vasculature to acute hypoxia, but it is not known whether exposure to sustained hyperoxia affects human pulmonary vascular control. We hypothesized that exposure to 8 h of hyperoxia would diminish the hypoxic pulmonary vasoconstriction (HPV) that occurs in response to a brief exposure to hypoxia. Eleven healthy volunteers were studied in a crossover protocol with randomization of order. Each volunteer was exposed to acute isocapnic hypoxia (end‐tidal $P_{O_{2}}$ = 50 mmHg for 10 min) before and after 8 h of hyperoxia (end‐tidal $P_{O_{2}}$ = 420 mmHg) or euoxia (end‐tidal $P_{O_{2}}$ = 100 mmHg). After at least 3 days, each volunteer returned and was exposed to the other condition. Systolic pulmonary artery pressure (an index of HPV) and cardiac output were measured, using Doppler echocardiography. Eight hours of hyperoxia had no effect on HPV or the response of cardiac output to acute hypoxia.

## Introduction

The human pulmonary circulation responds to alveolar hypoxia with an abrupt acute vasoconstriction occurring over a few (~5) minutes. If the hypoxia is maintained, this constriction begins to intensify after about 40 min (Talbot et al. 2005), and continues to increase over the periods of 2–8 h that have been studied in various laboratory protocols (Dorrington et al. 1997; Balanos et al. 2002; Talbot et al. 2014). This increasing vasoconstriction in the presence of a constant sustained hypoxic stimulus can be thought of as ‘pulmonary vascular acclimatization’ to hypoxia by analogy with the human ventilatory acclimatization to hypoxia that follows a similar time course. The term ‘hypoxic pulmonary vasoconstriction’ (HPV) is variably applied to both the acute and the longer term responses.

The question of whether the oxygen sensing of the pulmonary vasculature and that of the ventilatory control system share similarities in the manner in which they generate their respective acclimatizations to hypoxia continues to receive attention. This is of particular interest given the observation that both pulmonary vascular and ventilatory sensitivities to hypoxia are modulated by the hypoxia‐inducible factor (HIF) system of transcription regulation (Smith et al. 2006). In a recent study of 80 volunteers, each exposed to 8 h of isocapnic hypoxia (end‐tidal $P_{O_{2}}$, $P_{{\mathrm{ET}}_{O_{2}}}$ = 55 mmHg), the time courses of these two processes of acclimatization were found to be very similar, and the magnitudes of the acclimatization responses seen at 8 h represented more than a doubling of the early responses seen at 30 min of hypoxia (Fatemian et al. 2015). However, on assessing the variability between individuals with regard to the two responses, an absence of correlation was found. Indeed, a fundamental difference between the two processes of acclimatization was revealed by that study: the increase in sensitivity of the pulmonary vascular response to acute hypoxic challenges during the acclimatization was independent of the starting sensitivity on first exposure, whereas the increase in sensitivity of the ventilatory response to acute hypoxic challenges during the acclimatization was in proportion to the starting sensitivity on first exposure. This difference remains unexplained, and highlights our lack of a complete understanding of the underlying cellular pathways.

A feature of the pulmonary vascular and the ventilatory responses to oxygen that is of related interest is the extent to which ‘desensitization’ can be induced in both systems during a prolonged exposure to a high level of oxygen, namely to sustained hyperoxia. In 14 volunteers exposed to 8 h of isocapnic hyperoxia ($P_{{\mathrm{ET}}_{O_{2}}}$ = 300 mmHg), Ren and colleagues found a progressive decrease in ventilation over 8 h (after an initial anticipated (Becker et al. 1996) hyperventilatory response to hyperoxia), and a significant decrease of 16% in the sensitivity of the ventilatory response to brief periods of hypoxia during the 8‐h exposure (Ren et al. 2000). A similar phenomenon was shown during a 5‐day exposure to very modest hyperoxia ($P_{{\mathrm{ET}}_{O_{2}}}$ = normal value + 10 mmHg) (Donoghue et al. 2005). The human pulmonary circulation has recently been shown to respond to acute hyperoxia ($P_{{\mathrm{ET}}_{O_{2}}}$ = 100–175 mmHg) with a modest dilatation (Croft et al. 2013), but it is not known whether sustained hyperoxia is capable of inducing a progressive decrease in response, a desensitization analogous to that seen in the ventilatory control system. We hypothesized that exposure to 8 h of isocapnic hyperoxia would diminish the HPV that occurs in response to a brief (acute) exposure to hypoxia, thereby mirroring the increase in acute HPV that is brought about by prior exposure to 8 h of hypoxia.

## Methods

### Participants

Eleven healthy volunteers (10 men, one woman) were recruited to this study with a mean age of 22.5 years (SD = 3.4) and with a range of 18–27 years. Mean height and weight were 176.9 cm (SD = 5.4) and 67.9 kg (SD = 11.0). No volunteer had a history of respiratory, cardiac, or other diseases. All volunteers visited the laboratory in advance of participation to confirm detectability of tricuspid regurgitation with echocardiography and discuss the experiment requirements and details. On each experiment day, participants were requested to refrain from alcohol and drinks containing caffeine. The female participant underwent the experiment within the first 14 days of her menstrual cycle. Experiment information sheets were given in advance and written informed consent was obtained from each volunteer before participation. This study was approved by East Central London Research Ethics Committee 1 (Reference: 10/H0721/21) and was performed in accordance with the general ethical principles of the Declaration of Helsinki.

### Protocols

**Figure 1:**
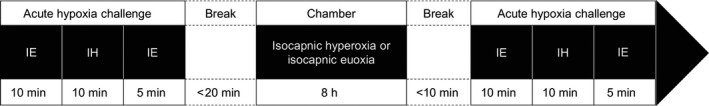
Schematic diagram of time‐course of the two protocols. IE, isocapnic euoxia; IH, isocapnic hypoxia. This was a cross‐over study, in which each volunteer experienced two conditions (hyperoxia and euoxia) on separate occasions.

Figure 1 is a schematic of the time course for the protocol. Each participant underwent two different 8‐h exposures in a customized chamber: (1) isocapnic hyperoxia, with $P_{{\mathrm{ET}}_{O_{2}}}$ held at 420 mmHg and end‐tidal $P_{{\mathrm{CO}}_{2}}$ ($P_{{\mathrm{ET}}_{{\mathrm{CO}}_{2}}}$) held at each volunteer's pre‐exposure value; and (2) isocapnic euoxia (control), in which the volunteer's $P_{{\mathrm{ET}}_{O_{2}}}$ was held at 100 mmHg and $P_{{\mathrm{ET}}_{{\mathrm{CO}}_{2}}}$ was held at each volunteer's pre‐exposure value. The sequence of exposures was randomized and concealed from each volunteer. At least 3 days separated each individual's two exposures to minimize any potential interactions. The technique for such 8‐h exposures to hyperoxia and control (euoxia) has been described previously (Ren et al. 2000). In short, volunteers wore a nasal catheter through which respired gas was constantly sampled, and inspired, and end‐tidal $P_{O_{2}}$ and $P_{{\mathrm{CO}}_{2}}$ were identified by computer on a breath‐by‐breath basis. The composition of chamber (inspired) gas was adjusted automatically every 5 min, or at manually overridden intervals, to bring the end‐tidal gases as close as possible to the target values.

### Measurements made before, during & after the 8‐h chamber exposures

The effects of sustained exposure were examined by measurements during the exposure itself and by acute hypoxic challenges before and after the exposure. During the 45 min before, and within 35 min following, each 8‐h exposure, the volunteer experienced an acute hypoxic challenge with three gas‐controlling steps: (1) isocapnic euoxia, with $P_{{\mathrm{ET}}_{O_{2}}}$ held at 100 mmHg and $P_{{\mathrm{ET}}_{{\mathrm{CO}}_{2}}}$ held at the volunteer's pre‐exposure value for 10 min; (2) isocapnic hypoxia, with peto_2_ held at 50 mmHg and $P_{{\mathrm{ET}}_{{\mathrm{CO}}_{2}}}$ held at the volunteer's pre‐exposure value for 10 min; (3) isocapnic euoxia, with $P_{{\mathrm{ET}}_{O_{2}}}$ and $P_{{\mathrm{ET}}_{{\mathrm{CO}}_{2}}}$ held as step 1 for 5 min. During the break of up to 10 min between leaving the chamber and starting the three gas‐controlling steps, the volunteer breathed room air. Our experience from previous studies has been that changes in pulmonary vascular sensitivity following an 8‐h exposure to hypoxia persist for one or more hours after finishing the exposure. For example, a study with pulmonary artery catheters found a persistent increase in sensitivity of the pulmonary vasculature to acute hypoxia present 3 h after a chamber exposure to 8 h of hypoxia (Dorrington et al. 1997). We therefore assumed that if a desensitization was produced by the 8‐h period of hyperoxia, it would not be ‘washed–out’ within that 20‐min period.

The technique for these acute exposures to 10‐min periods of hypoxia has been described previously (Robbins et al. 1982; Liu et al. 2013). In short, volunteers wore a nose clip and breathed through a mouthpiece, from which a catheter provided constant sampling of respired gas, and within which a turbine provided measurement of ventilation. Identification of inspired and end‐tidal $P_{O_{2}}$ and $P_{{\mathrm{CO}}_{2}}$ breath‐by‐breath was used to adjust the inspired gas automatically to bring the end tidal gases as close as possible to the target values.

Throughout the acute hypoxic exposures, each volunteer lay in the left lateral recumbent position as they breathed through a mouthpiece. During the hourly measurements in the chamber, each volunteer lay in the same left lateral recumbent position while breathing chamber gas. Systolic pulmonary artery pressure (SPAP), heart rate, stroke volume, and cardiac output were measured at minute intervals during the acute hypoxic challenges, and at hourly intervals during the 8‐h chamber exposures, using established echocardiographic techniques and equipment (Vivid i with 3S‐RS transducer, GE Healthcare; Vivid q with M4S‐RS transducer, GE Healthcare) (Smith et al. 2008). In short, SPAP is estimated from the pressure difference between the right ventricle and the right atrium (estimated using Bernoulli's equation from the peak velocity of a systolic tricuspid regurgitant jet obtained using continuous‐wave Doppler), to which was added an estimated right atrial pressure of 5 mmHg. The measurements obtained at time zero were called ‘pre‐exposure values’.

During both chamber exposures and acute hypoxic challenges, peripheral oxyhemoglobin saturation (SpO_2_) was measured, using a pulse oximeter (Ohmeda Biox 3740 Pulse Oximeter, BOC Healthcare) and a finger probe (OXY‐F4‐H Finger Sensor, GE Healthcare). The Ohmeda Biox 3740 uses two wavelengths for its detection of the pulsatile component of tissue/blood transmission: 660 nm (red) and 940 nm (infrared). The manufacturer states that carboxyhemoglobin may erroneously increase readings and that the increase is expected to be approximately equal to the amount of carboxyhemoglobin present. The manufacturer also states that methemoglobin tends to drive the reading toward 85%. Motion artifact would be a potential problem with this device, but we ensured volunteers remained very still during measurements. Heart rate was measured through a three‐lead electrocardiogram (Micromon 7142 B ECG, Kontron Medical).

Ventilation was measured during the acute hypoxic challenges by a turbine device (Ventilation Measurement Module, SensorMedics). All equipment was calibrated before each experiment.

### Statistical analyses

ANOVA was used to test for differences between the two 8‐h protocols. The Bonferroni procedure was used in the multiple comparisons (post hoc analysis) with variables during acute hypoxic challenges before and after the 8‐h exposures. The statistical significance level was set at 0.05. Values are mean ± SEM if not otherwise stated. The analysis was performed in IBM SPSS software package version 20.

## Results

All of the 11 volunteers completed the study, and none reported significant discomfort from the chamber exposures or acute hypoxic exposures.

### Changes during the 8‐h exposures to euoxia & hyperoxia

**Figure 2:**
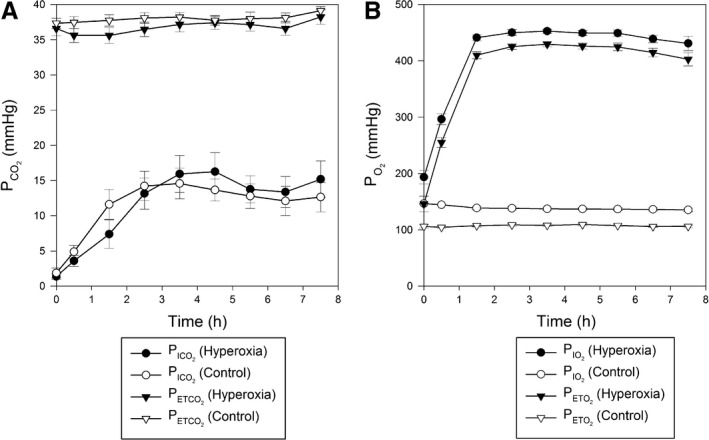
Control of end‐tidal and inspired gases in chamber. $P_{{\mathrm{CO}}_{2}}$, partial pressure of carbon dioxide, $P_{O{}_{2}}$, partial pressure of oxygen. (A) Gas control of inspired $P_{\mathrm{CO}{}_{2}}$, $P_{\mathrm{ICO}{}_{2}}$ (*circles*) and expired $P_{\mathrm{CO}{}_{2}}$, $P_{\mathrm{ET}{}_{\mathrm{CO}{}_{2}}}$ (*triangles*); (B) Control of inspired $P_{O{}_{2}}$, $P_{\mathrm{IO}{}_{2}}$ (*circles*) and expired $P_{O{}_{2}}$, $P_{\mathrm{ET}{}_{O{}_{2}}}$ (*triangles*). Data from hyperoxia and euoxia protocols are shown as *filled* and *empty* symbols, respectively. Values are means ± SEM.

Gas control during the chamber exposures is shown in Figure 2. In the hyperoxia protocol, volunteers' peto_2_ was increased to approximately 420 mmHg after 1.5 h in the chamber.

In the euoxia (control) protocol, volunteers' $P_{\mathrm{ET}{}_{O{}_{2}}}$ was maintained at 100 mmHg during 8 h in the chamber. In both protocols, the $P_{\mathrm{ET}{}_{\mathrm{CO}{}_{2}}}$ of each volunteer remained fairly constant during 8 h; there was a slight fluctuation during sustained hyperoxia.

**Figure 3:**
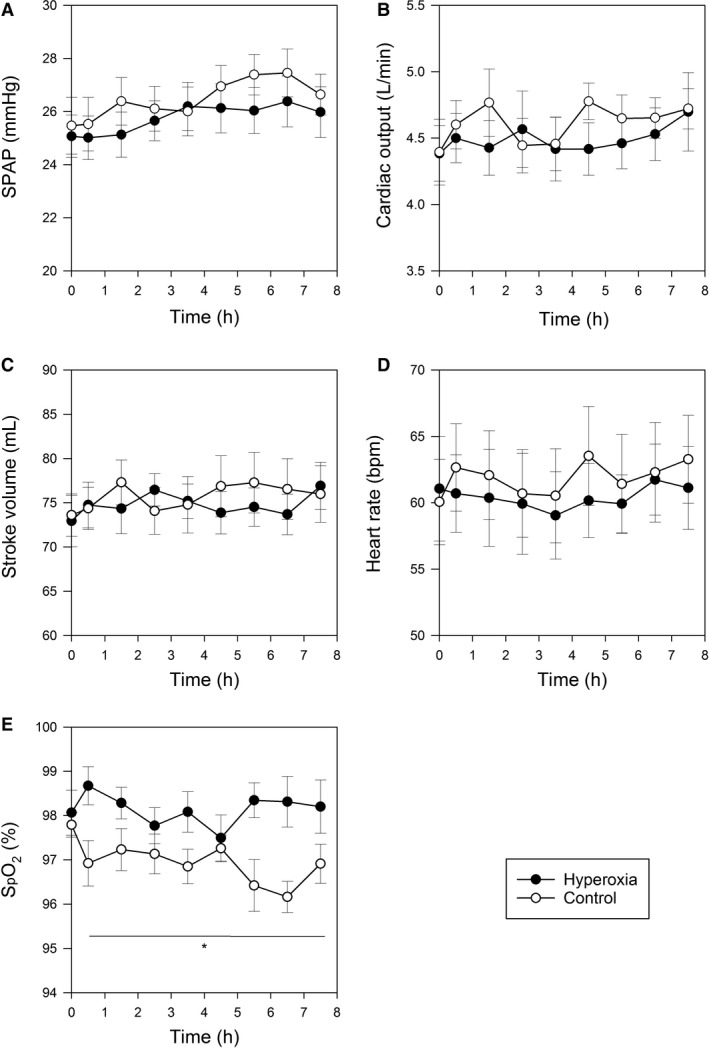
Cardiopulmonary responses during chamber exposures. (A) Systolic pulmonary artery pressure (SPAP); (B) Cardiac output; (C) Stroke volume; (D) Heart rate; (E) Oxyhemoglobin saturation (SpO_2_) were measured pre‐exposure (0‐h) and at hourly intervals in hyperoxia (*filled circles*) and euoxia (control, *empty circles*) exposures. Values are means ± SEM. *P < 0.05 when comparing protocols.

Cardiopulmonary responses to sustained hyperoxia are plotted in Figure 3. There was a significant difference between the hyperoxia exposure and the euoxia exposure in the values of SpO_2_ (Hyperoxia: 98.1 ± 0.4%; Control: 96.8 ± 0.3%, *P* = 0.016) (Fig. 3E). It should be noted that SpO_2_ was measured with volunteers in the left lateral recumbent position required for the simultaneous measurement of SPAP. It may well be that this posture contributes to the generation of a degree of dependent atelectasis that lowers the SpO_2_ to mean values somewhat below 100% during the breathing of approximately 60% oxygen. No significant differences were found in SPAP, cardiac output, stroke volume, or heart rate between the two conditions (Fig. 3A–D).

### Changes occurring during the acute hypoxic challenges before and after 8‐h exposures

**Figure 4:**
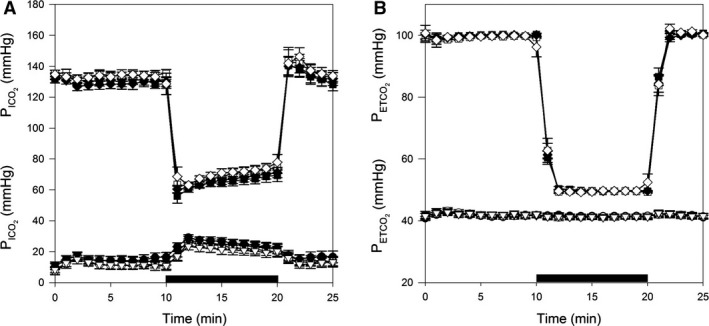
Control of end‐tidal (E) and inspired (I) gases during acute hypoxic exposures before and after chamber. (A) Control of $P_{\mathrm{ICO}{}_{2}}$ (prior: *circle*; post: *triangle*) and $P_{\mathrm{IO}{}_{2}}$ (prior: *square*; post: *diamond*); (B) Control of $P_{\mathrm{ET}{}_{\mathrm{CO}{}_{2}}}$ (prior: *circle*; post: *triangle*) and $P_{\mathrm{ET}{}_{O{}_{2}}}$ (prior: *square*; post: *diamond*) were averaged at 30‐sec intervals during acute hypoxic exposures before and after the chamber exposure. Data from hyperoxia and euoxia (control) protocols are shown as *filled* and *empty* symbols, respectively. The black bar shows the period of isocapnic hypoxia. Values are means ± SEM.

**Figure 5:**
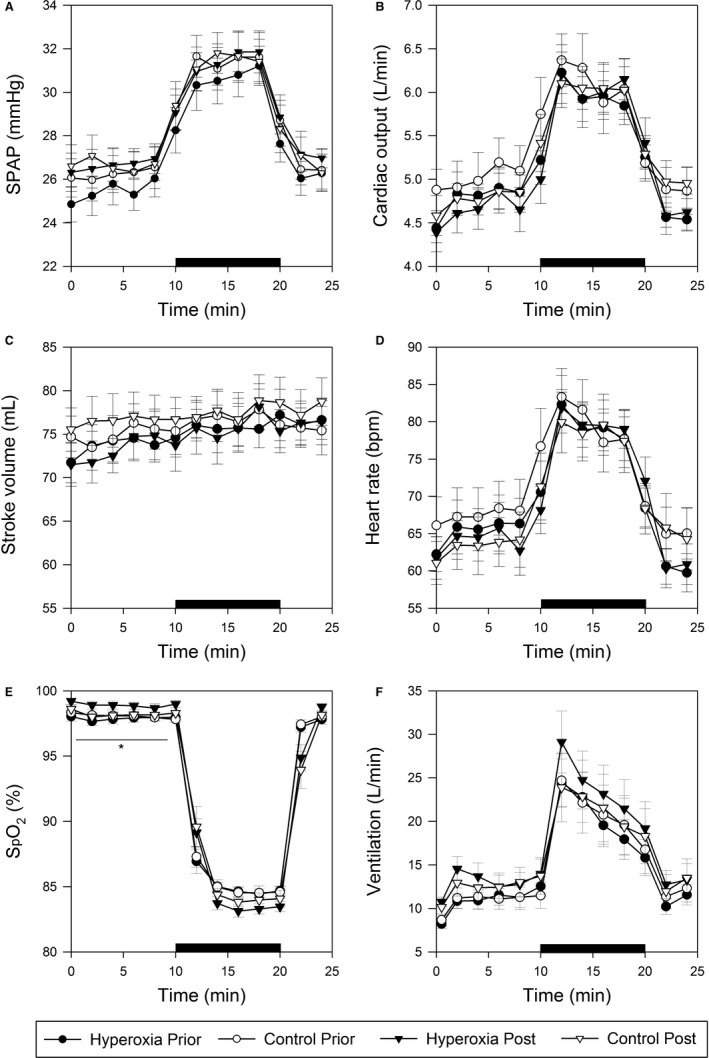
Changes in cardiopulmonary responses during acute hypoxic exposure before and after the chamber exposure. (A) Systolic pulmonary artery pressure (SPAP); (B) Cardiac output; (C) Stroke volume; (D) Heart rate; (E) Oxyhemoglobin saturation (SpO_2_). Data from hyperoxia (*filled* symbols) and euoxia (control, *empty* symbols) protocols are labeled by symbol filling; data from prior to (*circles*) and post (*triangles*) 8‐h exposures are labeled by symbol shape. The black bar shows the period of isocapnic hypoxia. Values are means ± SEM. *P < 0.05 when comparing prior to (97.9 ± 0.3%) and post (98.9 ± 0.3%) hyperoxia exposures.

Gas control during the acute hypoxic challenges prior to and postchamber exposures is shown in Figure 4. $P_{\mathrm{ET}{}_{O{}_{2}}}$ and $P_{\mathrm{ET}{}_{\mathrm{CO}{}_{2}}}$ remained stable during acute hypoxic challenges. Cardiopulmonary responses during acute hypoxic challenges prior to and postchamber exposures are shown in Figure 5. The SPAP values during acute hypoxic challenges prior to and post‐8‐hour exposures are similar in both exposures (Fig. 5A). No differences were found between the hyperoxia and control protocols with regard to cardiac output, stroke volume, or heart rate (Fig. 5B–D).

After the 8‐h hyperoxic exposure, SpO_2_ during isocapnic euoxia was higher than before the exposure (hyperoxia prior: 97.9 ± 0.3%; hyperoxia post: 98.9 ± 0.3%, *P* = 0.045) (Fig. 5E). No equivalent significant difference was found in the control protocol. Although ventilation after the 8‐h hyperoxia exposures was higher than ventilation before the exposure, the difference was not statistically significant (Fig. 5F). We note that a slightly lower SpO_2_ during acute hypoxia following the 8 h of hyperoxia may suggest an increase in the mismatch between perfusion and ventilation. If this were the case, one might expect a lower SpO_2_ during the euoxic phase of these tests; interestingly the opposite was found to be the case, with the SpO_2_ being significantly higher.

## Discussion

The main finding of this study is the absence of any pulmonary vascular desensitization to hypoxia during an exposure to isocapnic hyperoxia lasting 8 h. We had hypothesized, drawing parallels with ventilatory acclimatization to hypoxia and desensitization to hyperoxia, that exposure to 8 h of hyperoxia would diminish the HPV that occurs in response to a brief exposure to hypoxia. In Figure 5A, it can be seen that no difference in the acute response of SPAP to 10 min of isocapnic hypoxia was detectable following the 8‐h hyperoxic exposure, when compared with control experiments. Furthermore, the absence of any effect of 8 h of hyperoxia on the response of cardiac output to acute hypoxia helps to ensure that no subtle pulmonary vascular effect of the hyperoxia is masked by some difference in flow through the pulmonary vascular system in the experiments shown in Figure 5.

A related indication that no change in the acute HPV response to changes in peto_2_ developed during the 8‐h hyperoxic exposure is that SPAP itself did not differ from the euoxic control during the 8 h of hyperoxia, in contrast to the gradual rise in SPAP measured during an 8‐h exposure to isocapnic hypoxia (Talbot et al. 2014; Fatemian et al. 2015).

### Limitations in the study

This study was not designed to resolve a ventilatory desensitization of the kind observed by Ren et al. (2000) who studied volunteers exposed to 8 h of eucapnic hyperoxia at the level of $P_{\mathrm{ET}{}_{O{}_{2}}}$ = 300 mmHg. In that study, acute ventilatory sensitivity to eucapnic hypoxia was measured, using sequences of six 1‐min exposures to hypoxia, from which dynamic modeling detected a fall in sensitivity G_p_ of 16% following the 8‐h exposure to hyperoxia. In the present study, a 10‐min exposure to acute hypoxia was used to study the acute pulmonary vascular response, during which measurements of ventilation showed no significant difference from control or baseline (Fig. 5F). Notwithstanding the difference between the techniques used, the explanation for this difference between the findings of the two studies is unclear. One possibility is that the significantly higher peripheral oxyhemoglobin saturation during the euoxic 10‐min run into the acute hypoxic exposure (Fig. 5E) may have been associated with a greater excursion in the arterial po_2_ seen by the chemoreflex afferents in the carotid body and masked an effect detected in the earlier study, using different methodology. These observations suggest that it might be of benefit, in studies of this kind, to be able to sample arterial blood from an indwelling systemic arterial cannula in order to be able to assess the extent to which manipulation of $P_{\mathrm{ET}{}_{O{}_{2}}}$ achieves similar values of arterial $P_{O{}_{2}}$.

### Comparison with acute effects of hyperoxia in a previous study

A small nonsignificant difference in SPAP between the euoxia and hyperoxia protocols of ~1 mmHg can be seen in Figure 3A during the 8‐h periods of chamber exposure. During the exposure, there was a significant difference in of SpO_2_ observed of 1.3%. The question arises as to whether these findings are consistent with those seen in an earlier study of the acute effects of isocapnic hyperoxia on the human pulmonary circulation in exposures lasting 4–10 min (Croft et al. 2013). In that study, the sensitivity of SPAP to changes in SpO_2_ was found to be 0.43 ± 0.09 mmHg/% desaturation. For a difference in SpO_2_ of 1.3%, this would predict an acute fall in SPAP of 0.56 mmHg, consistent with the observation made in the current study of 8‐h hyperoxia as being outside the resolution available here. A new finding from the present 8‐h exposures is that no observable gradual change in SPAP is found to be induced by hyperoxia comparable to the progressive elevation in SPAP observed between 1 and 8 h during isocapnic hypoxia in the same setting, using the same methodology (Balanos et al. 2005; Talbot et al. 2014); in these studies, the degree of acclimatization was commonly as great as, or greater than, the response occurring within the first hour.

### Cellular mechanisms of oxygen sensing involving HIF

A body of evidence suggests that both pulmonary vascular and ventilatory acclimatization to hypoxia are determined, at least in part, by the HIF regulators of transcription (Smith et al. 2006), and that differences between the two may be associated with different localizations or other aspects of the components of this system. The processes of pulmonary vascular and ventilatory acclimatization are associated with an increase in the acute sensitivities, respectively, of the pulmonary vasculature and the ventilatory system to acute episodes of hypoxia. Some studies have used these changes in acute sensitivities to assess the role of the HIF system. Thus, for example, patients with Chuvash polycythemia have a generalized upregulation of the HIF system, and display both enhanced pulmonary vascular sensitivity and enhanced ventilatory sensitivity to acute hypoxia, as well as a dramatic degree of pulmonary arterial hypertension during the 6 h of modest hypoxia associated with a long‐distance flight (Smith et al. 2006, 2013). Patients with a gain‐of‐function mutation in HIF‐2*α* show an enhanced pulmonary vascular sensitivity to moderate acute hypoxia in the presence of a normal ventilatory sensitivity to the same stimulus (Formenti et al. 2011). In relation to a reduction in acclimatization in humans, healthy Tibetans living at sea level display a smaller degree of pulmonary vascular acclimatization to hypoxia than Han Chinese controls, and this is associated with a hyporesponsive HIF‐2 transcriptional system (Petousi et al. 2014).

In mice, Hodson and colleagues have recently shown that inducible inactivation of HIF‐2*α*, but not HIF‐1*α*, strikingly reduced ventilatory acclimatization to hypoxia and associated carotid body cell proliferation (Hodson et al. 2016). For pulmonary vascular acclimatization to hypoxia to occur in mice, Kapitsinou et al. (2016) have shown that it is the HIF‐2*α* localized to the endothelium that appears to be required, and that the linked expression of the vasoconstrictor peptide endothelin‐1 may provide the mechanism of vascular smooth muscle constriction.

These studies raise the question of whether the differing effects of oxygen on ventilatory and pulmonary vascular control reflect tissue‐specific differences in cellular HIF regulation.

### Clinical implications of exposure to hyperoxia

The nature of responses to hyperoxia also carries a clinical relevance. Patients in hospital, divers, and professionals in the aerospace environment are often exposed to sustained periods of hyperoxia, and some evidence is accruing that this may lead to harmful physiological responses. This evidence has tended to be of two kinds. First, physiological changes have been observed during hyperoxia that are presumed to be potentially harmful to tissues and organs, such as reductions in regional blood flow and reductions in cardiac output (Rousseau et al. 2005; Orbegozo Cortés et al. 2015; Spoelstra‐de Man et al. 2015). Second, some studies on patient populations have demonstrated higher morbidity or mortality in groups experiencing higher levels of arterial oxygenation (Rincon et al. 2014; Helmerhorst et al. 2015, 2017; Girardis et al. 2016; Asfar et al. 2017).

Several studies have observed a decrease in cardiac output during hyperoxia, in contrast to the absence of a change observed here (Harten et al. 2003; Waring et al. 2003; Spoelstra‐de Man et al. 2015). Changes in $P_{\mathrm{ET}{}_{\mathrm{CO}{}_{2}}}$ are to be anticipated during hyperoxia as ventilation changes (Dripps and Comroe 1947; Becker et al. 1996) and have been confirmed during hyperoxia in association with hypocapnia (Van De Water et al. 1970; Rousseau et al. 2005; Weaver et al. 2009). (The current view of why ventilation acutely rises during hyperoxia is that the reduction in the Haldane effect renders the venous cerebral blood acidic and this stimulates central chemoreceptors.) Furthermore, isolated lowering of $P_{\mathrm{ET}{}_{\mathrm{CO}{}_{2}}}$ itself leads to a fall in cardiac output (Balanos et al. 2003). It thus usually remains unclear to what extent the physiological circulatory changes seen during hyperoxia can be attributed to changes in pco_2_ in blood rather than oxygen levels themselves. Similar considerations apply to measurements of pulmonary artery pressure during poikilocapnic hyperoxia (Van De Water et al. 1970; Weaver et al. 2009). The present study found no evidence of a fall in cardiac output with isocapnic hyperoxia held at a level of $P_{\mathrm{ET}{}_{O{}_{2}}}$ of 420 mmHg for 8 h.

## Conflict of Interest

None declared.
